# Simvastatin Treatment Upregulates HO-1 in Patients with Abdominal Aortic Aneurysm but Independently of Nrf2

**DOI:** 10.1155/2018/2028936

**Published:** 2018-03-20

**Authors:** Aleksandra Piechota-Polanczyk, Aleksandra Kopacz, Damian Kloska, Branislav Zagrapan, Christoph Neumayer, Anna Grochot-Przeczek, Ihor Huk, Christine Brostjan, Jozef Dulak, Alicja Jozkowicz

**Affiliations:** ^1^Department of Medical Biotechnology, Faculty of Biochemistry, Biophysics and Biotechnology, Jagiellonian University, Krakow, Poland; ^2^Department of Surgery, Division of Vascular Surgery, Medical University of Vienna, Vienna, Austria

## Abstract

Heme oxygenase-1 (HO-1), encoded by *HMOX1* gene and regulated by Nrf2 transcription factor, is a cytoprotective enzyme. Its deficiency may exacerbate abdominal aortic aneurysm (AAA) development, which is also often associated with hyperlipidemia. Beneficial effects of statins, the broadly used antilipidemic drugs, were attributed to modulation of Nrf2/HO-1 axis. However, the effect of statins on Nrf2/HO-1 pathway in patients with AAA has not been studied yet. We analyzed AAA tissue from patients treated with simvastatin (*N* = 28) or without statins (*N* = 14). Simvastatin treatment increased HO-1 protein level in AAA, both in endothelial cells (ECs) and in smooth muscle cells (SMCs), but increased Nrf2 localization was restricted only to vasa vasorum. Nrf2 target genes *HMOX1*, *NQO1*, and *GCLM* expression remained unchanged in AAA. *In vitro* studies showed that simvastatin raises HO-1 protein level slightly in ECs and to much higher extent in SMCs, which is not related to Nrf2/ARE activation, although *HMOX1* expression is upregulated by simvastatin in both cell types. In conclusion, simvastatin-induced modulation of HO-1 level in ECs and SMCs *in vitro* is not related to Nrf2/ARE activity. Likewise, divergent HO-1 and Nrf2 localization together with stable expression of Nrf2 target genes, including *HMOX1*, in AAA tissue denotes Nrf2 independency.

## 1. Introduction

Abdominal aortic aneurysm (AAA) is characterized by overproduction of free radicals and depletion of antioxidative enzymes which localization may vary depending on the aortic layer and the stage of aneurysm development. Yajima et al. [1] indicated that during AAA development in rodents, over 200 genes involved in oxidative stress are upregulated including heme oxygenase-1 (*HMOX1*), inducible nitric oxide synthase (*NOS2*), or 12*-lipoxygenase* (*ALOX12*). Recent studies of Ho et al. [2] presented that mice lacking HO-1 are more prone to angiotensin II-induced AAA with more severe elastin degradation, medial degeneration, increased macrophage recruitment, and matrix metalloproteinase- (MMP-) 9 level. Also, Azuma et al. [3] showed that HO-1 heterozygote mice have higher concentration of proinflammatory cytokines in blood such as monocyte chemoattractant protein-1 (MCP-1), tumor necrosis factor-*α* (TNF-*α*), interleukin- (IL-) 1*β*, and IL-6, which emphasizes the anti-inflammatory role of HO-1. Those authors also reported that HO-1 induction by heme slows down AAA progression. Other protective effects of HO-1 may include reduction of vascular smooth muscle cell (VSMC) proliferation, inhibition of platelet aggregation, and attenuation of vasoconstriction [4, 5].

HO-1 is an inducible enzyme which catalyzes oxidative degradation of heme to equimolar amounts of carbon monoxide (CO), biliverdin, and ferrous iron [6]. The control of HO-1 expression occurs primarily at the transcriptional level and is mediated by different transcription factors such as nuclear factor kappa B (NF-*κ*B) and nuclear factor E2-related factor-2 (Nrf2) [7]. However, our recent studies indicated that HO-1 expression may be regulated not only at the mRNA level but also at the protein level by increased ubiquitination and proteasomal degradation of HO-1 and that this effect is independent of Nrf2 [8].

Under normal conditions, Nrf2 remains in the cytoplasm in a complex with Keap1. Upon stress conditions, Nrf2 is released from the inhibitory complex and translocates to the nucleus leading to the activation of antioxidant response element- (ARE-) mediated gene expressions. Nrf2 binding to its consensus sequence ARE is forerun by dissociation of Bach1 from ARE and its relocation to the cytoplasm [9, 10]. Activation of Nrf2 may have a protective role in VSMC due to induction of antioxidative genes such as *HMOX1*, NAD(P)H quinone dehydrogenase 1 (*NQO1*), or glutamate-cysteine ligase modifier subunit (*GCLM*) and decrease in synthesis of proinflammatory mediators [11]. It was recently shown that antioxidant ursodeoxycholic acid prevents acute aortic dissection via activation of Nrf2 and Nrf2-regulated antioxidant redox enzymes in aortic VSMC [12]. Furthermore, Nrf2 activation that leads to the higher expression of *HMOX1* is regulated by oxidative stress and can be augmented by therapeutic agents such as statins [13, 14].

Statins, inhibitors of 3-hydroxy-3-methylglutaryl-coenzyme A (HMG-CoA) reductase, decrease the conversion of HMG-CoA to L-mevalonate and coenzyme A. They improve endothelial cell function, modify inflammatory response, reduce VSMC proliferation, and attenuate cholesterol accumulation by reducing concentration of low-density lipoprotein (LDL), triglyceride-rich lipoproteins, and nonsteroidal isoprenoid compounds in plasma [15–17]. Our previous reports showed that patients treated with simvastatin had decreased oxidative stress, reduced proinflammatory TNF-*α* level, changed concentration of matrix metalloproteinase- (MMP-) 2, MMP-9, and tissue inhibitors of MMPs (TIMPs), and attenuated activity of proinflammatory mediators such as NF-*κ*B and extracellular signal regulated kinases (ERK) 1/2 [18–20]. Furthermore, statins target circulating neutrophil gelatinase-associated lipocalin (NGAL) and MMP-9/NGAL, the biomarkers of cardiovascular diseases [21]. It was also presented that statins may activate antiapoptotic protein kinase B- (Akt/PKB-) related signaling pathways, which increase *HMOX1* resulting in optimal human aortic SMC cytoprotection [22]. Moreover, five-day simvastatin treatment (120 mg/kg/day) of rats triggers the nuclear translocation of Nrf2 in the liver and enhances Nrf2 recruitment to its binding sites on DNA, including ARE sequence in *HMOX1* gene promoter [23]. Statins may also suppress atrial tachypacing-induced cellular remodeling via the activation of Akt/Nrf2/HO-1 [24] and inhibit angiotensin II-induced VSMC inflammation by activation of Nrf2-dependent genes *NQO1* and *HMOX1* [25]. Therefore, simvastatin may influence *HMOX1* expression via Nrf2 in inflammatory-related diseases. However, its influence on Nrf2/HO-1 has not been studied in patients with AAA, yet.

The aim of this study was to verify the localization of HO-1 and Nrf2 in human AAA wall and to analyze the influence of simvastatin treatment on Nrf2/ARE system and Nrf2-related genes in AAA wall as well as in cells composing aortic wall: aortic endothelial cells and smooth muscle cells.

## 2. Material and Methods

### 2.1. Patients

This study comprised 59 patients who underwent open AAA repair between September 2009 and December 2011 at the Department of Surgery, Medical University of Vienna, according to our previously described analysis [18]. We chose patients treated only with simvastatin or who had taken no statins for at least 6 months before the AAA repair and matched them by AAA diameter and age. Finally, 42 patients were selected and divided into 14 “nonstatin” patients (10 men and 4 women) and 28 simvastatin-treated patients (25 men and 3 women) to study the effects of simvastatin on HO-1 and Nrf2 in AAA. The treated group took 20 mg to 40 mg of simvastatin daily (according to body weight, liver enzymes, and blood lipids) for a minimum of 6 months. The exclusion criteria included (1) taking statins other than simvastatin and nonsteroidal anti-inflammatory drugs, except aspirin in the medication list; (2) chronic diseases such as liver, inflammatory, and malignant diseases; (3) recreational drug intake; and (4) alcohol abuse. All patients signed written informed consent before data and sample collection.

Aneurysm wall tissue was harvested during surgery for retrospective analysis. AAA diameter was measured with preoperative computed tomography angiography.

The study was approved by the local institutional ethics committee (EC 294/2009) at the Medical University of Vienna.

### 2.2. Tissue Harvesting and Sample Processing

After aortic cross-clamping and longitudinal incision of the aneurysm, thrombus was removed and about 3 cm^2^ of the aneurysm sack at the site of its maximum diameter was excised. Aneurysm wall samples were cut in half and placed in 10% formalin or immediately frozen in liquid nitrogen and stored at −80°C. For subsequent biochemical analyses, aneurysmal tissues were cut into 50 mg pieces and rinsed with ice-cold saline to eliminate liquid components such as blood and residual thrombi. Tissue processing was always conducted on ice to avoid tissue degradation. Samples in formalin were further paraffinized for histological analyses.

### 2.3. *In Vitro* Experiments on Primary Human Aortic Endothelial Cells and Aortic Smooth Muscle Cells

Primary human aortic endothelial cells (HAoEC) isolated from 67-year-old Caucasian male (Gibco) were cultured in EBM-2 medium with 10% FBS and supplements (EGM 2MV, Lonza). Before stimulation, cells were starved for 24 h with EBM-2 with 0.5% of FBS and streptomycin/penicillin.

Primary human aortic smooth muscle cells (HAoSMC) isolated from 54-year-old Caucasian male (Gibco) were cultured in M231 medium with 10% FBS and supplements (Gibco). Before stimulation, cells were starved for 24 h with M231 with 0.5% of FBS and streptomycin/penicillin.

As AAA samples came from elder patients, we used the cells between passages 8 and 18. HAoEC or HAoSMC were stimulated with activated simvastatin at the dose of 1 and 10 *μ*M for 6 h and/or 24 h to analyze changes in gene expression and protein level. Simvastatin (Sigma-Aldrich) was activated using the protocol previously described by Dong et al. [26]. Simvastatin doses which did not influence cell viability, measured with 3-(4,5-dimethylthiazol-2-yl)-2,5-diphenyltetrazolium bromide (MTT) assay, were chosen for experiments [27].

### 2.4. Verification of the Role of Nrf2 in Simvastatin-Induced Changes of HO-1

To analyze Nrf2 localization, cells were stimulated with simvastatin (1 and 10 *μ*M) or sulforaphane (SFN; 10 *μ*M) for 1 h, fixed with absolute methanol, and stained as described below.

To analyze Nrf2 role in simvastatin-induced upregulation of HO-1 in HAoEC and HAoSMC, we diminished Nrf2 expression using siRNA and transduced cells with Ad-Nrf2-DN (encoding transcriptionally inactive Nrf2), respectively. In the first experiments, HAoECs were transfected with 50 nM siRNA targeted against human NFE2L2 (Nrf2) or scrambled siRNA (Life Technologies; cat. number s9493) using Lipofectamine™ 2000 Transfection Reagent for HAoEC (Life Technologies) and Lipofectamine RNAiMAX for HAoSMC (Life Technologies) in Opti-MEM I Reduced Serum medium (Life Technologies). 48 h after transfection, cells were starved, stimulated with simvastatin (1 and 10 *μ*M) for 6 h, and collected for RNA isolation (described below).

In the second experiments, HAoECs or HAoSMC were transduced with adenoviral vectors Ad-Nrf2-DN (encoding transcriptionally inactive Nrf2) or Ad-GFP at the 50 multiplicity of infection (MOI) dose. The efficiency of transduction was confirmed by detection of GFP expression with the fluorescent microscope and qPCR assessment of Nrf2 target genes. At 48 h after transduction, cells were starved and stimulated with simvastatin (1 and 10 *μ*M) or sulforaphane (SFN; 10 *μ*M) for 6 h after which cells were collected for RNA isolation.

Transcriptional activity of Nrf2 was performed on HAoECs cotransfected with 0.4 *μ*g of plasmid ARE-luc containing the sequence ARE driving the expression of luciferase and with 0.1 *μ*g of the pCMV-LacZ plasmid containing the *β*-galactosidase gene driven by the CMV promoter (Promega) using Lipofectamine 2000 Transfection Reagent (Life Technologies) as previously described [28]. At 48 h after transfection, cells were stimulated with simvastatin (1 and 10 *μ*M) or sulforaphane (SFN; 10 *μ*M) for 6 h. Luciferase activity was quantified using the Luciferase Assay System (Promega), according to the manufacturer's protocols. Luminescence was measured for a period of 10 seconds in Tecan Spectra II Microplate Reader (Tecan). *β*-Galactosidase activity was measured using the *β*-Galactosidase Enzyme Assay with Reporter Lysis Buffer (Promega). Absorbance was measured at 420 nm using Tecan Spectra II Microplate Reader (Tecan).

### 2.5. Immunohistochemical Staining of Nrf2 and HO-1 in AAA Samples and Immunofluorescent Staining of Nrf2 in Cells

Paraffinized samples were cut to 5 *μ*m slices using microtome (Thermo Fisher Scientific), deparaffinized and boiled for 15 min in citric acid buffer (pH 6.0) to activate antigen, permeabilized in 0.01% of Triton X-100 for 2 min, washed, and incubated with 0.25% of glycine in PBS for 30 min. Next, tissue scraps were blocked for 1 h in 0.5% of goat serum (GS) at room temperature. After washing in PBS, samples were incubated overnight (4°C) with anti-rabbit Nrf2 polyclonal antibodies (dilution 1 : 50 H-300, Santa Cruz) diluted in 0.5% GS. At the next day, samples for Nrf2 IHC were washed and incubated with anti-rabbit HRP-conjugated antibody for 1 h at room temperature and the reaction was visualized with DAB substrate kit (Abcam). Nuclei were counterstained with haematoxylin. Samples were analyzed under a light microscope (Nikon).

Frozen samples were cut to 5 *μ*m slices using cryostat (Leica), permeabilized in 0.01% of Triton X-100 for 2 min, washed, and incubated with 0.25% of glycine in PBS for 30 min. Next, tissue scraps were blocked in 0.5% of goat serum (GS) (for Nrf2) or in 10% bovine serum albumin (BSA) with 0.05% Tween 20 (for HO-1, von Willebrand factor (vWF) or myosin smooth muscle heavy chain (SMV)) for 1 h at room temperature. After washing in PBS, samples were incubated overnight (4°C) with rabbit anti-Nrf2 polyclonal antibodies (dilution 1 : 100; H-300, Santa Cruz), rabbit anti-HO-1 polyclonal antibodies (dilution 1 : 100; Enzo SPA894), mouse anti-vWF (1 : 250; Abcam), and mouse anti-myosin smooth muscle heavy chain (SMV clone N1/5; dilution 1 : 400; Sigma-Aldrich) diluted in either 0.5% GS or 1% BSA. On the next day, samples were washed and incubated with anti-rabbit antibodies conjugated with Alexa Fluor 488 (dilution 1 : 1000; IgG H + L, Life Technologies) or anti-mouse antibodies conjugated with Alexa Fluor 568 (dilution 1 : 1000; IgG, Life Technologies) for 1 h at RT. Nuclei were counterstained with Hoechst 33342 (dilution 1 : 10,000) during the second washing. Samples were analyzed under a fluorescent microscope (Nikon).

For immunofluorescence staining, HAoECs and HAoSMC were washed in PBS, fixed (in 4% PFA for HO-1 and Bach1 and methanol for Nrf2 and tubulin), and washed 3 times in PBS. Afterwards, the cells were incubated in 0.25% glycine in PBS solution for the next 30 minutes at room temperature and washed 3 times in PBS, then blocked in 3% BSA in PBS for 1 h at room temperature. The cells were probed with primary antibody for HO-1 (dilution 1 : 100; SPA 894, Enzo), Bach1 (dilution 1 : 100, Santa Cruz Biotechnology), Nrf2 (1 : 100, Santa Cruz Biotechnology, H-300), and tubulin (dilution 1 : 500, Calbiochem) in 3% BSA in PBS, overnight at 4°C. On the next day, cells were 3 times washed in PBS and incubated with secondary antibodies conjugated with Alexa Fluor 488 or Alexa Fluor 568 (dilution 1 : 1000, Life Technologies). The cells were stained with Hoechst 33342 (dilution 1 : 10,000) to visualize nuclei. High-resolution images were taken using a fluorescent microscope (Nikon) or a metalaser scanning confocal microscope (LSM-510; Carl Zeiss).

### 2.6. Analysis of *HMOX1*, *NQO1*, *GCLM*, and *NFE2L2* Gene Expressions by Real-Time Quantitative Polymerase Chain Reaction (RT-qPCR)

RNA from 30 mg of aortic tissue or from cultured cells was extracted with RNeasy Mini Kit (Qiagen, Germany) according to the manufacturer's instructions. cDNA was synthesized using High-Capacity cDNA Reverse Transcription Kit (Thermo Fisher Scientific, USA). RT-qPCR was conducted on StepOne Plus Real-Time PCR Systems using a Power SYBR® Green PCR Master Mix according to the manufacturer's instructions (Thermo Fisher Scientific). Primer sequences are gathered in Table 1. Eukaryotic translation elongation factor 2 (*hEF-2*) was used as a reference gene. The results are presented as ΔCT for tissue or ΔCT and fold change for cells. All experiments were run in three independent replicates.

### 2.7. Assay of Glutathione in Tissue Samples

Enzymatic colorimetric analysis for assessment of total and oxidized glutathione level in tissue samples was performed following a protocol described by Giustarini et al. [29] with further modifications. Briefly, aortic wall samples were homogenized in Tris-BSAN buffer (50 mM Tris buffer with serine/boric acid/acivicin/NEM; pH 8.0), acidified with 60% trichloroacetic acid, and centrifuged (14,000 ×g, 2 min, room temperature). Total GSH was measured in supernatants after adding 5-5′-dithiobis[2-nitrobenzoic acid] (DTNB), *β*-nicotinamide adenine dinucleotide phosphate (NADPH), and glutathione reductase. Changes in absorbance were monitored for 5 minutes at *λ* = 412 nm. Total glutathione was calculated from calibration curve prepared from increasing values of reduced glutathione standards (10, 25, 50, 75, and 100 *μ*M). Oxidized GSSG was analyzed in the same sample after extraction of N-ethylmaleimide (NEM) with dichloromethane (DCM). Reduced GSH was calculated by subtraction of GSSG form total GSH. The results were presented as *μ*M per mg of tissue. Experiments were done in duplicates.

### 2.8. Analysis of HO-1 and Nrf2 Protein Expressions by Western Blot and Enzyme-Linked Immunosorbent Assays (ELISA)

Aortic wall samples or cells were homogenized in ice-cold RIPA buffer containing protease and phosphatase inhibitors. After 30 min incubation on ice and centrifugation (8000 ×g, 10 min, 4°C), supernatant was collected and protein concentration was assayed using previously described bicinchoninic acid protein assay method [30]. A total of 30 *μ*g of protein were separated electrophoretically and transferred to nitrocellulose membrane (0.45 *μ*m) by wet transfer (Bio-Rad Laboratories). After blocking in 5% nonfat milk for 1 h, membranes were incubated overnight (4°C) with primary rabbit antibody against Nrf2 (Cell Signaling) (dilution 1 : 500), rabbit anti-HO-1 polyclonal antibodies (dilution 1 : 1000; Enzo), or mouse anti-*β*-tubulin antibody as a reference (dilution 1 : 1000; Merck Millipore). Next, membranes were washed with Tris-buffered saline-Tween 20 (TBST) buffer and incubated for 1 h with secondary antibodies conjugated with HRP: anti-rabbit IgG (Cell Signaling) and anti-mouse IgG (BD) (dilutions 1 : 5000). Following three washes in TBST buffer, the bands were visualized using SuperSignal HRP Substrate (Merck Millipore) and X-ray films. Densitometric analysis of protein expression levels was conducted using ImageJ version 1.51f software (http://rsbinfo.nih.gov/ij/; USA). The results were calculated as the ratio of HO-1 and Nrf2 over *β*-tubulin expression. All experiments were conducted in triplicate.

HO-1 protein concentration was determined in tissue lysates using human HO-1 ELISA kit (ADI-EKS-800, Enzo) following the manufacturer's protocol. 1 g of tissue was lysed in 1 ml of extraction reagent from the kit. Samples were diluted twice in reaction buffer before assay. The concentration of HO-1 was read from calibration curve and presented as ng/ml of tissue lysate. Samples were measured in duplicates.

### 2.9. Statistical Analysis

Continuous demographic and biochemical data are presented as median, minimum, and maximum or mean ± SE; demographic categorical data were described with absolute frequencies and percentages. Comparisons between groups were performed using the Student *t*-test, Kruskal-Wallis test (or nonparametric Mann–Whitney *U* test), and *χ*^2^ test. Two-way analysis of variance and the Dunn's posttest were used to calculate differences depending on the normality of distribution. To calculate correlations, Spearman's rank correlation coefficient (*r*) test was used. Grubbs' test was performed to calculate statistically significant outliers (*p* < 0.05), which were not included in statistical analysis of the results (GraphPad Prism software).

## 3. Results

### 3.1. Patient Characteristic

The characteristic of examined groups is presented in Table 2. The aneurysm diameter did not differ significantly between the analyzed groups (median 55 mm (49–102 mm) in the nonstatin group and 56.0 mm (49–120 mm) in the simvastatin group). Patients on simvastatin had significantly lower total cholesterol and LDL cholesterol levels compared to the nonstatin group (*p* = 0.009 and *p* = 0.007, resp.). No differences in C-reactive protein (CRP), fibrinogen, creatinine, hemoglobin, or leukocyte levels were noticed (all *p* ≥ 0.05).

### 3.2. Effect of Simvastatin on HO-1 and Nrf2 Level, Expression of Nrf2-Regulated Genes, and Redox Status in AAA Wall

Activated Nrf2 translocates to the nucleus and stimulates expression of target genes such as *HMOX1*, *NQO1*, and *GCLM*, which in turn regulate cell antioxidant capacity by influencing the production and scavenging rate of reactive oxygen species (ROS) or affecting the glutathione metabolism [31]. HO-1 is an antioxidative enzyme whose expression is regulated by inflammatory- or oxidative stress-related signaling pathways, not only by Nrf2 but also by NF-*κ*B or AP2 [32].

**Figure 1:**
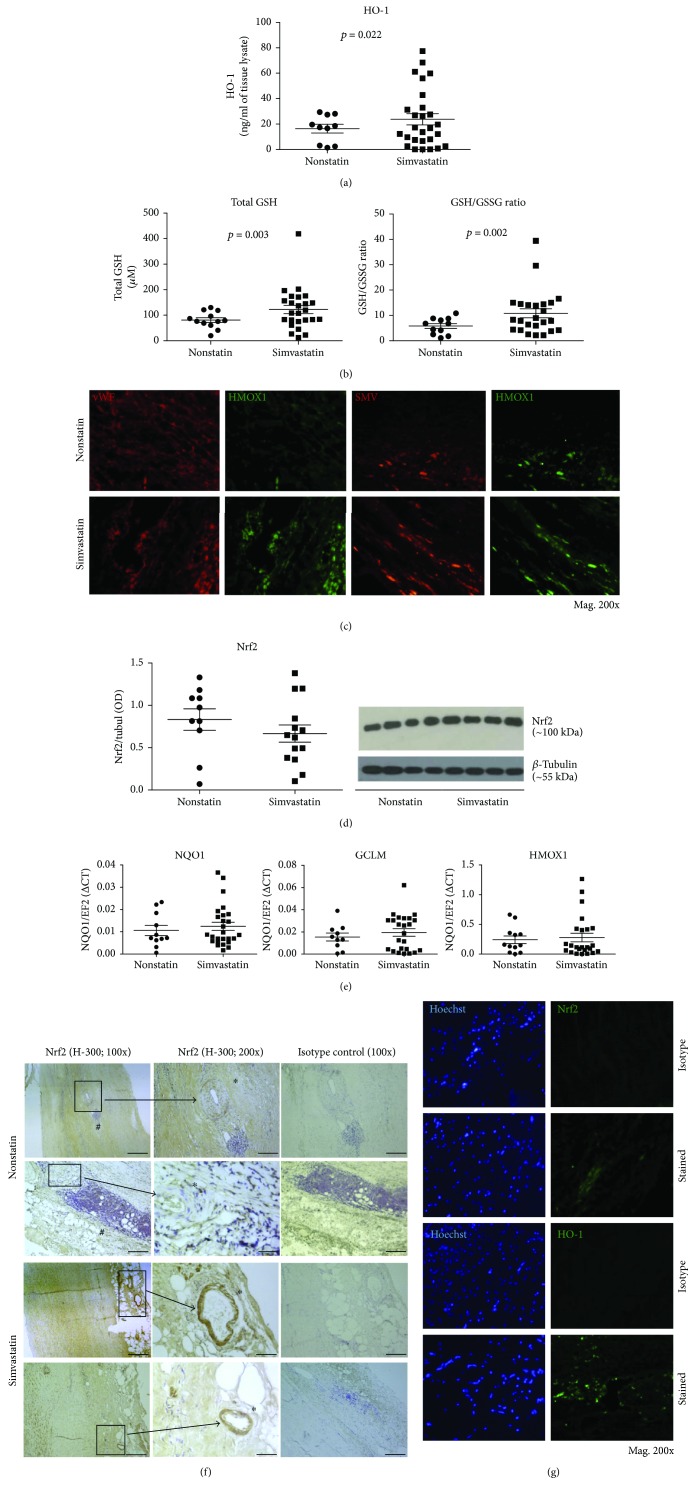
Simvastatin treatment increases HO-1 protein, but not mRNA level in AAA. (a) Protein level of HO-1 (measured with ELISA) in aneurysm wall of nonstatin and simvastatin-treated patients. (b) Changes in total glutathione and the ratio of GSH/GSSG in aortic aneurysm wall. (c) Immunofluorescent staining of HO-1 in ECs and SMCs in aortic aneurysm wall. HO-1, heme oxygenase 1; Nrf2, nuclear factor E2-related factor-2; vWF, von Willebrand factor (EC marker); SMV, myosin smooth muscle heavy chain (SMC marker). (d) Nrf2 level in AAA tissue. Densitometry data and representative blot. (e) mRNA levels of Nrf2-dependent genes: *NQO*1, *GCLM*, and *HMOX1* in AAA wall. (f) Localization of Nrf2 in AAA wall. The brown color represents positive staining (IHC). Vasa vasorum is indicated by asterisks (^∗^). Inflammatory infiltration indicated by hash (#) was stronger in the nonstatin patient. (g) Illustrates the distribution of Nrf2 and HO-1 staining (IF) within AAA tissue. Average data from panels a, b, d, and e represents mean ± SE.

It was previously shown that simvastatin exerts its protective effect on vascular system through induction of HO-1 [33]. Therefore, we first analyzed HO-1 protein level within AAA tissue in patients treated with simvastatin and in untreated patients. As determined by ELISA, the protein level of HO-1 in tissue extracts was higher in the simvastatin-treated patients compared to the untreated group (23.68 ± 4.42 versus 16.37 ± 3.40 ng/ml, resp.; *p* = 0.022; Figure 1(a)). As HO-1 is an antioxidative enzyme, its upregulation might affect cellular redox status. Therefore, we analyzed the influence of simvastatin on total, reduced, and oxidized GSH in AAA wall (Figure 1(b)). The results indicated that simvastatin-treated patients had significantly higher level of total GSH (122.3 ± 16.45 versus 80.96 ± 9.45 *μ*M in the simvastatin versus control; *p* = 0.003; Figure 1(b)). This was accompanied with markedly increased GSH/GSSG ratio in AAA wall of the simvastatin-treated group in comparison to the nonstatin (10.82 ± 1.79 versus 5.80 ± 0.97, resp.; *p* = 0.002; Figure 1(b)). Interestingly, total GSH positively correlated with HO-1 protein concentration (rho = 0.35, *p* = 0.041, Spearman's rank correlation test), which might indicate the role of HO-1 in maintenance of redox status in AAA wall. Then, we aimed to determine the localization of HO-1 in AAA wall. We observed that HO-1 is expressed in whole tissue (primarily endothelial cells and smooth muscle cells) and its expression is upregulated in patients treated with simvastatin, at least in EC (Figure 1(c)).

As both HO-1 and glutathione are regulated by Nrf2, taking the next step, we aimed to verify the effect of statins on the level of Nrf2 and its main targets. Analysis of tissue lysates indicated a comparable level of Nrf2 protein in both groups (Figure 1(d)). What is more, there were no significant changes in the mRNA levels of Nrf2 target genes: *HMOX1*, *NQO1*, and *GCLM* within AAA tissue in patients treated with simvastatin and in untreated patients (Figure 1(e)). We found a statistically significant positive correlation between expression of *HMOX1* and *NQO1* (rho = 0.46, *p* < 0.01), *GCLM* and *NQO1* (rho = 0.50, *p* < 0.01), and *HMOX1* and *GCLM* (rho = 0.68, *p* < 0.001) in patients, as analyzed by Spearman's rank correlation test. All these results indicate that simvastatin treatment does not influence Nrf2 transcriptional activity in AAA wall. Interestingly, the expression of *HMOX1* at mRNA level did not correlate with concentration of HO-1 protein (rho = −0.09, *p* = 0.56, Spearman's rank correlation test). The discrepancy between mRNA and protein level of HO-1 might indicate an additional, posttransciptional regulation of HO-1 in this case.

Furthermore, histological staining showed that expression of Nrf2 and HO-1 is not homogenous in the vessel wall and could be regulated locally. We observed that simvastatin-treated patients had a higher local expression of Nrf2 protein in aneurysmal wall, especially in vasa vasorum (Figure 1(f)). What is more, we noticed different distribution of HO-1 and Nrf2 in the AAA wall (Figure 1(g)), which indicates Nrf2 independency.

### 3.3. Simvastatin Leads to Upregulation of *HMOX1* mRNA Expression in HAoEC and HAoSMC That Is Not Associated with Nrf2/ARE Axis

Immunohistochemical staining and ELISA indicated that Nrf2 and HO-1 proteins can be locally upregulated within AAA wall in the simvastatin-treated patients, mainly in endothelial and smooth muscle cells (Figures 1(a), 1(c), 1(f), and 1(g)). Therefore, we checked if the response to simvastatin could be observed in primary HAoEC and HAoSMC. We used *in vitro* cell cultures to detect the direct cellular effects of simvastatin and exclude those resulting from modulation of metabolism at the organismal level [20].

**Figure 2:**
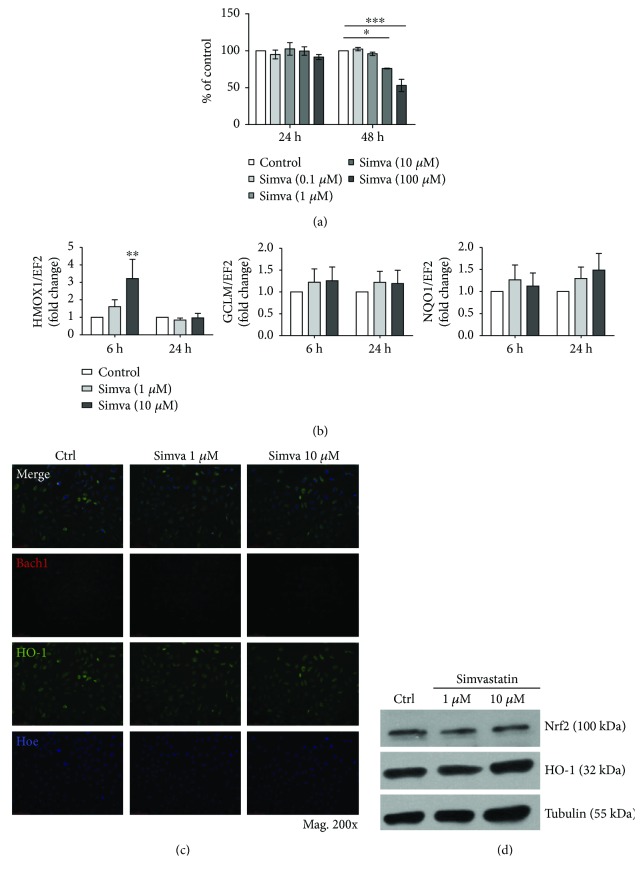
Simvastatin upregulates *HMOX1* mRNA and slightly increases HO-1 protein in HAoEC. (a) Simvastatin influence on HAoEC viability after 24–48 h of stimulation (MTT assay). (b) mRNA level of *HMOX1*, *GCLM*, and *NQO1* in HAoEC after simvastatin treatment (1 and 10 *μ*M) for 6 h and 24 h. (c) Immunofluorescent staining of HO-1 (green) and Bach1 (red) in HAoEC after stimulation with simvastatin (1 and 10 *μ*M) for 6 h. (d) Protein level of HO-1 and Nrf2 after 24 h stimulation with simvastatin (1 and 10 *μ*M) in representative Western blot. Data are presented as mean ± SE; ^∗^*p* < 0.05, ^∗∗^*p* < 0.01, and ^∗∗∗^*p* < 0.001 (*N* = 3‐4).

First, we verified the influence of simvastatin on HAoEC viability. Here, we showed that simvastatin at concentrations of 0.1, 1, and 10 *μ*M did not inhibit growth of cultured cells up to 24 h. However, some decrease in HAoEC proliferation was noticed after 48 h (Figure 2(a)). Therefore, concentrations of 1 and 10 *μ*M were chosen for further tests. What is more, basing on the literature data, such concentrations of simvastatin increased HO-1 expression in human ECs [33] and human RPE cells [34]. Also, 10 *μ*M of simvastatin was shown to correspond with simvastatin concentration in human serum (2.2–4.3 nM) when a patient is treated with 40 mg simvastatin, which was the common dose in our simvastatin-treated group of patients [35, 36].

As half-life for statins is between 0.7 and 3 hours [37], we stimulated HAoEC for 6 h or 24 h. The results indicated that simvastatin might have a transient and early effect on *HMOX1*, as an increase in *HMOX1* mRNA after 6 h of incubation was noticed (Figure 2(b)). However, we observed that HO-1 at the protein level remained rather stable with some tendency to increase (Figures 2(b)–2(d)). Moreover, *GCLM* and *NQO1*, as well as Bach1, a signaling molecule that dissociates from ARE when Nrf2 binds to DNA, remained rather stable (Figures 2(b) and 2(c)). This may indicate that the effect of simvastatin on *HMOX1* mRNA is not strictly followed by the level of HO-1 protein and that simvastatin has no direct influence on Nrf2 transcriptional activity in HAoEC. However, the weak and transient effect observed in cultured cells may also suggest that the influence of simvastatin on HO-1 protein level in AAA tissue could have been associated with modulation of metabolism at the organismal level, for example, changes in cholesterol or LDL cholesterol level (Table 1), rather than with direct effect on Nrf2 expression or transcriptional activity.

**Figure 3:**
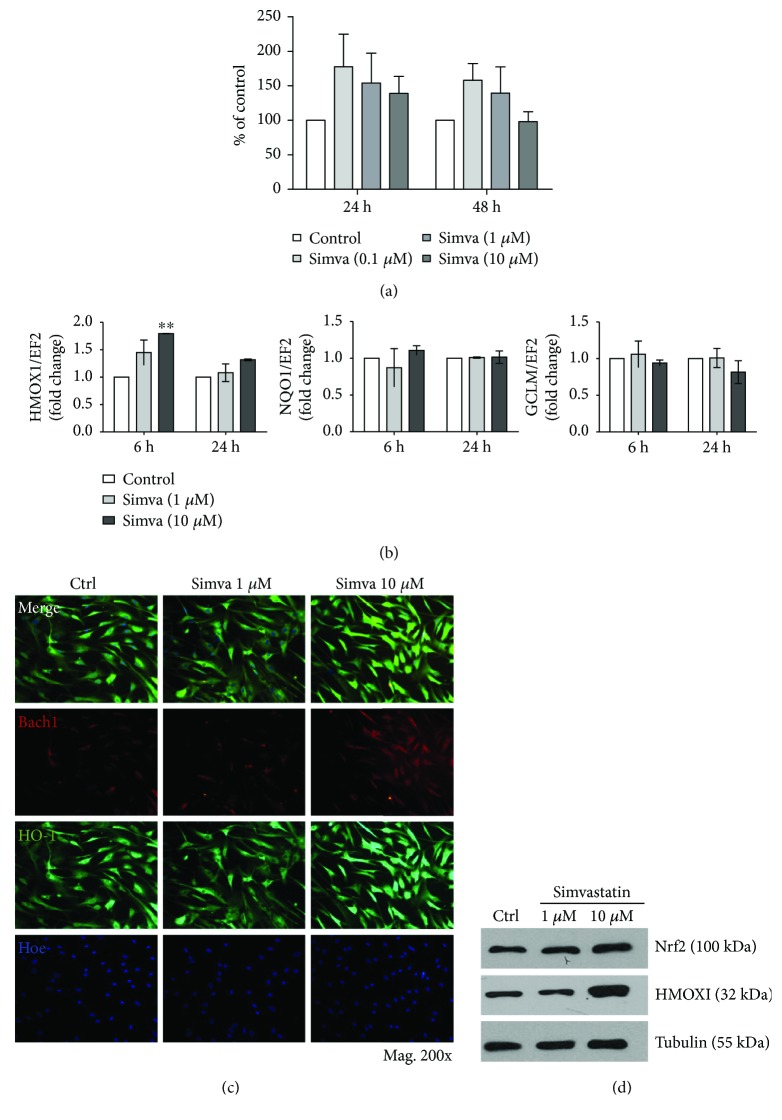
Simvastatin upregulates *HMOX1* mRNA and increases HO-1 protein in HAoSMC. (a) Simvastatin influence on HAoSMC viability after 24–48 h of stimulation (MTT assay). (b) mRNA level of *HMOX1*, *GCLM*, and *NQO1* in HAoSMC after simvastatin treatment (1 *μ*M and 10 *μ*M) for 6 h and 24 h. (c) Immunofluorescent staining of HO-1 (green) and Bach1 (red) in HAoSMC after stimulation with simvastatin (1 and 10 *μ*M) for 6 h. (d) Protein expression of HO-1 and Nrf2 after 24 h stimulation with simvastatin (1 and 10 *μ*M) presented in representative Western blot. Data are presented as mean ± SE. ^∗∗^*p* < 0.01 (*N* = 3‐4).

As simvastatin-treated patients had upregulated Nrf2 level in vasa vasorum (Figure 1(f)), we next studied its influence on HAoSMC. First, we confirmed that the tested doses of simvastatin (0.1, 1, and 10 *μ*M) did not influence the viability of HAoSMC, although some toxicity of the highest dose after 48 h incubation was noticed (Figure 3(a)). Therefore, we chose 1 and 10 *μ*M doses for further experiments.

The results indicated that simvastatin might have an effect on HO-1 as upregulation of *HMOX1* mRNA (Figure 3(b)) and a strong increase in HO-1 at the protein level (Figures 3(c)-3(d)) with an increase in Bach1, a signaling molecule that dissociates from ARE when Nrf2 binds to DNA, was observed (Figures 3(b)-3(c)). However, *GCLM* and *NQO1* remained stable (Figure 3(b)).

Finally, we wanted to verify if simvastatin-induced changes in *HMOX1* gene and protein are dependent on Nrf2/ARE system. Our results indicated that simvastatin at higher doses (10 *μ*M) led to significant upregulation of *HMOX1* at the mRNA in both HAoEC and HAoSMC (Figures 2(b) and 3(b), resp.). However, Western blot results showed that higher dose of simvastatin has slight effect on HO-1 protein level in HAoEC (Figure 2(d)) and it increases HO-1 level in HAoSMC (Figure 3(d)). Interestingly, Nrf2 protein level was not influenced by simvastatin in both cell lines (Figures 2(d) and 3(d)); however, an upregulation of Bach1 in the cytoplasm was observed in HAoSMC (Figure 3(c)). Therefore, having such cell type-dependent results on the protein expression of HO-1, with simultaneous increase in *HMOX1* gene in both cell lines, we decided to further analyze the subject and to verify the possibility of Nrf2/ARE-dependent increase of *HMOX1* after simvastatin treatment.

**Figure 4:**
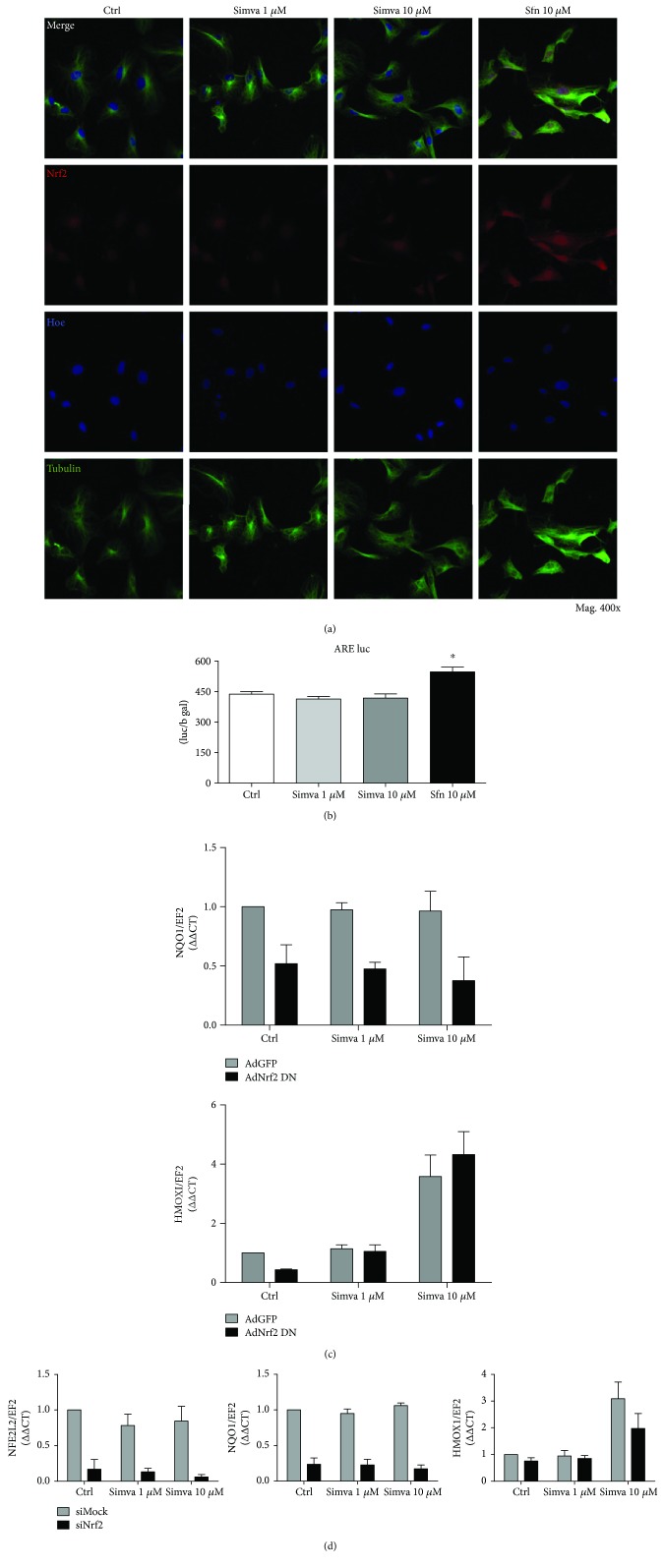
Simvastatin does not influence *HMOX1* via Nrf2/ARE system in HAoEC. (a) Nrf2 localization after 1 h stimulation with simvastatin (1 and 10 *μ*M) or sulforaphane (10 *μ*M; positive control). Confocal microscopy: Nrf2 (red), nuclei (blue), and tubulin (green) (Mag. 400x). (b) *β*-Galactosidase activity measured with luciferase reported assay of HAoEC transfected with the ARE-dependent luciferase gene plasmid and stimulated for 6 h with simvastatin (1 and 10 *μ*M) or sulforaphane (10 *μ*M, positive control). (c) Changes in gene expression of *NQO1* and *HMOX1* in HAoEC with transcriptionally inactive Nrf2 (Ad-Nrf2-DN) stimulated for 6 h with simvastatin (1 and 10 *μ*M). (d) Efficiency of Nrf2 silencing in HAoEC transfection with siNFE2L2 (Nrf2) and changes in expression of *NQO1* and *HMOX1* after 6 h stimulation with simvastatin (1 and 10 *μ*M). Data are presented as mean ± SE; ^∗^*p* < 0.05, ^∗∗^*p* < 0.01, and ^∗∗∗^*p* < 0.001 versus siNFE2L2/AdGFP; ^$^*p* < 0.05 versus simva 1 *μ*M siNFE2L2 (*N* = 3).

**Figure 5:**
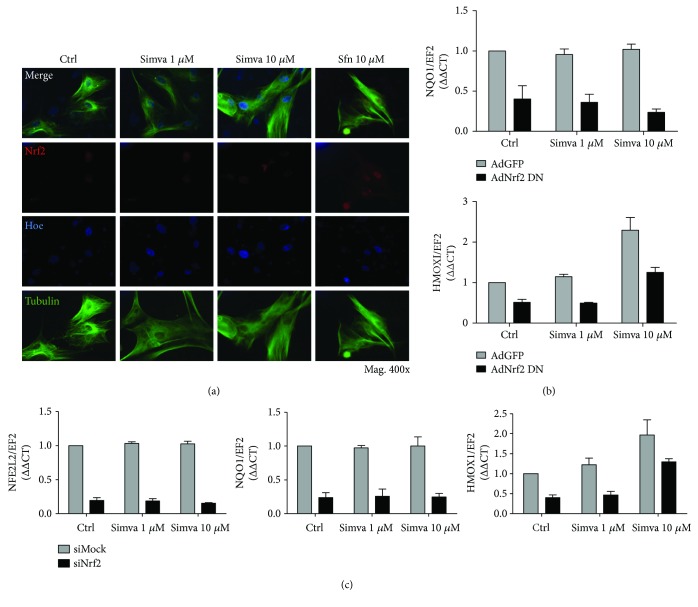
Simvastatin does not influence *HMOX1* via Nrf2/ARE system in HAoSMC. (a) Nrf2 localization after 1 h stimulation with simvastatin (1 and 10 *μ*M) or sulforaphane (10 *μ*M, positive control). Confocal microscopy: Nrf2 (red), nuclei (blue), and tubulin (green) (Mag. 400x). (b) Changes in gene expression of *NQO1* and *HMOX1* in HAoSMC with transcriptionally inactive Nrf2 (Ad-Nrf2-DN) stimulated for 6 h with simvastatin (1 and 10 *μ*M). (c) Efficiency of Nrf2 silencing in HAoSMC transfection with siNFE2L2 (Nrf2) and changes in expression of *NQO1* and *HMOX1* after 6 h stimulation with simvastatin (1 and 10 *μ*M). Data are presented as mean ± SE; ^∗^*p* < 0.05, ^∗∗^*p* < 0.01, and ^∗∗∗^*p* < 0.001 versus siNFE2L2/AdGFP; ^#^*p* < 0.05 versus siNFE2L2/AdGFP control; ^$$^*p* < 0.01 versus simva 1 *μ*M siNFE2L2 (*N* = 3).

First, to verify if simvastatin leads to nuclear translocation of Nrf2, we stimulated cells with simvastatin or sulforaphane for 1 h and checked Nrf2 localization. The results indicated no significant increase in Nrf2 translocation to the nucleus after simvastatin compared to the control. However, treatment with SFN led to an increase in Nrf2 protein in cytoplasm and nucleus in both cell lines (Figures 4(a) and 5(a)).

As HO-1 expression may be modulated by Nrf2 transcriptional activity, we transfected cells with ARE-luc plasmid, encoding luciferase under control of promoter containing Nrf2 consensus sequence. The reporter assay showed that simvastatin, unlike sulforaphane, did not induce transcription of luciferase driven by ARE promoter, thus indicating no influence of simvastatin on Nrf2 transcriptional activity in HAoEC (Figure 4(b)). Moreover, we found no effect of simvastatin on *HMOX1* expression in HAoEC and HAoSMC after transduction of cells with transcriptionally inactive Nrf2 (Ad-Nrf2-DN) (Figures 4(c) and 5(b), resp.). It is worth pointing out that in both groups, we observe around 2-fold induction of *HMOX1* gene. Also, despite successful silencing of Nrf2, confirmed by decreased expression of *NFE2L2* gene in siNFE2L2-treated cells, we did not observe any influence of Nrf2 silencing on induction of *HMOX1* upon stimulation with simvastatin (Figures 4(d) and 5(c)). The level of *NQO1* was not affected by treatment with simvastatin in any examined groups.

Therefore, we conclude that simvastatin-induced upregulation of HO-1 in HAoEC or HAoSMC is not associated with Nrf2/ARE system and simvastatin-induced upregulation of *HMOX1* gene with simultaneous downregulation of HO-1 at the protein level might be associated other mechanisms such as posttransciptional regulation of HO-1 as it was shown in our recently published paper [8].

## 4. Discussion

Our results give insights into expression of *HMOX1* and Nrf2 within AAA tissue in relation to statin therapy. We found that simvastatin-treated patients have upregulated HO-1 in EC and SMC as well as higher GSH/GSSG ratio in aneurysmal wall. Both HO-1 and GSH are regulated by Nrf2 in aortic cells [38, 39]. However, we noticed increased Nrf2 localization only in vasa vasorum, and no change in the expression of Nrf2 targeted genes suggesting that HO-1 in AAA tissue is not directly regulated by Nrf2.

Additionally, we demonstrated that distribution of HO-1 and Nrf2 is not homogenous within AAA wall. Both proteins were highly expressed in the media layer, but expression was not equally distributed. Previously, Ishizaka et al. [40] showed that in normal murine aorta, HO-1 is localized to the medial SMC and adventitial cells but not to EC; however, during pathological conditions, like hypertension, HO-1 expression increases especially in adventitial and endothelial cells. Furthermore, a study on vascular injury in mice demonstrated that Nrf2 level may increase in apoptotic cells in the middle stages of neointimal expansion [41]. This suggests that Nrf2-dependent genes may also be elevated in those regions.

We also found that simvastatin treatment can upregulate HO-1 protein in AAA tissue, but as Nrf2 was only higher in vasa vasorum, and taking into account lack of effect on NQO1 level, we assume the regulation is Nrf2 independent. The possible physiological importance of HO-1 upregulation after simvastatin treatment may be associated with HO-1 modulation of the vascular tone (via CO production) and the increasing antioxidative capacity of the tissue, for example, through elevated GSH synthesis [40, 42]. Moreover, it was presented that other statins like rosuvastatin may induce HO-1 in aortic tissue and suppress AAA progression [3]. In addition, rosuvastatin also acts protectively against atrial fibrillation via the activation of Akt/Nrf2/HO-1 signaling [24]. Another statin, atorvastatin, downregulates NF-*κ*B, promotes Nrf2 activity, and upregulates *NQO1* and *GCLC* in HAoSMC subjected to oxidative stress induced by angiotensin II [25]. Our previous reports also showed that AAA patients treated with simvastatin have lower oxidative stress and reduced NF-*κ*B and ERK1/2 signaling pathways [19, 20], thereby supporting the antioxidative role of simvastatin. The increased level of HO-1 protein observed in this study may suggest a higher antioxidative capacity of AAA tissue of simvastatin-treated subjects. The increased level of total GSH and GSH/GSSG ratio and a positive correlation between tissue HO-1 and total GSH concentration further support this hypothesis.

On the contrary, recently published data pointed out that upregulation of cholesterol caused oxidative damage in vascular EC and increased the expression of HO-1 via the activation of Nrf2 and the MAPK/ERK signaling pathway. Therefore, overexpression of HO-1 may alleviate oxidative damage [43]. Following this mechanism, simvastatin-induced decrease in total and LDL cholesterol levels of our AAA patients may partially explain the weak effects of simvastatin on HO-1 and Nrf2.

As there was a visible cell-dependent distribution of HO-1 and Nrf2 in subjects treated with simvastatin, we further verified the effect of simvastatin on those proteins in cultured HAoEC and HAoSMC. With this approach, we could detect the direct cellular effects of simvastatin and exclude those resulting from modulation of metabolism at the organismal level. The results indicated a transient upregulation of *HMOX1* after simvastatin both in HAoEC and HAoSMC. This effect, however, seemed to be independent of Nrf2 activation as we did not observe any translocation of Nrf2 to the nucleus or increase in Nrf2/ARE activity after simvastatin treatment. Moreover, silencing of Nrf2 with siRNA or silencing of Nrf2 transcriptional activity with adenoviruses did not alter *NQO1* but 2-fold induction on *mRNA* level was maintained despite Nrf2 inhibition. Thus, we hypothesize that there is an additional regulation of HO-1 protein level. We cannot exclude that in patients with AAA, simvastatin influences ubiquitination and proteasomal degradation of HO-1, therefore regulating its expression directly on the protein level and independently of Nrf2 which we recently shown in human and murine cells by our group [8]. Moreover, as aneurysmal tissue is infiltrated by inflammatory cells, the effect of simvastatin may be masked. Additionally, our experiments were performed on cells stimulated solely with simvastatin and without oxidative stress, induced, for example, by angiotensin II. Therefore, we cannot exclude that under stress conditions, effects of simvastatin on HO-1 and Nrf2 could differ from our results as it was indicated by others [39, 44]. However, Lee et al. [33] reported that simvastatin did not change HO-1 in cultured endothelial cells or macrophages but it increases HO-1 level in SMC *in vivo*. The increase of HO-1 in SMC was associated with activation of phosphoinositide 3-kinase (PI3K) and Akt pathway [33]. Additionally, Loboda et al. reported that atorvastatin has a weak and transient effect on HO-1 expression in human microvascular EC [45]. However, simvastatin has been described to increase HO-1 in human and rat umbilical ECs, but not in mice or bovine ECs [33, 46]. Consequently, the effect of simvastatin on HO-1 may depend on species, cell types, or cell culture conditions.

In conclusion, we presented that simvastatin-induced modulation of HO-1 level in ECs and SMCs *in vitro* is not related to Nrf2/ARE *HMOX1* transactivation. Increased HO-1 and GSH levels in aneurysmal tissue of simvastatin-treated patients were not associated with higher Nrf2 expression. Therefore, divergent HO-1 and Nrf2 localization together with stable expression of Nrf2 target genes, including *HMOX1*, in AAA tissue denote Nrf2 independency.

### 4.1. Limitation to the Study

It should be stated that primary cell, especially HAoEC, response to stimulation highly depends on donor's age and concomitant diseases. We noticed that cells isolated from elder donors have senescent phenotype and impaired response to proangiogenic stimulators such as SDF-1 (data not shown). Donor medical history, as is also of importance as primary cells isolated from donors with metabolic diseases such as diabetes mellitus, may have highly impaired proliferation. Therefore, primary cell response to stimulation is not only dose- and time-dependent but may be as well affected by the age of donors from which primary cells were isolated.

Furthermore, we observed that simvastatin influenced HO-1 gene and protein expression in HAoEC and HAoSMC. However, we did not perform functional tests related to HO-1 influence on EC or SMC proliferation as well as anti-inflammatory and antioxidant effects. Nevertheless, biological function of HO-1 regarding proliferation of endothelial and smooth muscle cells was thoroughly studied by our group in the past. We found that the polymorphism of *HMOX1* gene significantly modulates a cytoprotective, proangiogenic, and anti-inflammatory function of HO-1 in human endothelium [42]. We also showed that HO-1 is necessary for a proper proangiogenic function of bone marrow-derived cells [47] and that mice injected with medium from murine myoblasts (C2C12 cells) overexpressing HO-1 improved angiogenesis in the hind limb after ischemia-reperfusion probably via increasing stromal cell-derived factor- (SDF-) 1*α* [48]. Moreover, activation of HO-1 augmented myoblast proliferation and improves their viability under oxidative stress [49]. Also, chemical activation of HO-1 with hemin increased vascular endothelial growth factor (VEGF) production in human microvascular EC [50] and keratinocytes [51]. It was also suggested that angiogenic effects of hypoxia-inducible factor 1 in EC were associated with HO-1 overexpression by Nrf2. However, HO-1 upregulation did not influence the expression of an important angiogenic mediator, IL-8 [52]. Finally, we also presented that activation of HO-1 with tin protoporphyrin in rat VSMC improved cell viability, reduced production of VEGF, and increased expression of iNOS [53, 54].

## Figures and Tables

**Table 1:** Primer sequence.

Primer	Sequence
*HMOX1*	Forward: 5′ TTCTTCACCTTCCCCAACATTG 3′ Reverse: 5′ CAGCTCCTGCAACTCCTCAAA 3′
*NQO1*	Forward: 5′ AGGACCCTTCCGGAGTAAGA 3′ Reverse: 5′ CCAGGATTTGAATTCGGGCG 3′
*GCLM*	Forward: 5′ ACAGCGAGGAGCTTCATGAT 3′ Reverse: 5′ TGTGCAACTCCAAGGACTGA 3′
*NFE2L2*	Forward: 5′ TTGAGCAAGTTTGGGAGGAGCTA 3′ Reverse: 5′ GGAGAGGATGCTGCTGAAGG 3′
*hEF2*	Forward: 5′ GAGATCCAGTGTCCAGAGCAG 3′ Reverse: 5′ CTCGTTGACGGGCAGATAGG 3′

**Table 2:** Patient characteristic.

	Nonstatin	Simvastatin (20 or 40 mg/day)	*p* value
	*N* = 14	*N* = 28	
Age (range)	70 (50–80)	66 (50–80)	0.48
AAA diameter (mm) (range)	55.0 (49.0–102.0)	56.0 (49.0–120.0)	0.70
Body mass index (kg/m^2^) (range)	25.62 (21.97–37.55)	25.95 (20–34.6)	0.80
Coronary artery disease	7/14 (50%)	7/28 (25%)	0.31
Cerebrovascular artery disease	7/14 (50%)	4/28 (14%)	0.77
Hypertension	13/14 (93%)	14/28 (50%)	0.28
Peripheral artery disease	2/14 (14%)	7/28 (25%)	0.20
Diabetes	2/14 (14%)	4/28 (14%)	0.34
Nicotine	8/14 (57%)	11/28 (39%)	0.80
CRP (mg/dl) median (range)	0.40 (0.04–3.0)	0.26 (0.06–9.45)	0.75
Cholesterol (mg/ml) median (range)	240 (143.0–323.0)	206.5 (110.0–300.0)	***0.009***
HDL (mg/ml) median (range)	46.0 (32.0–68.0)	47.0 (29.0–75.0)	0.61
LDL (mg/dl) median (range)	159.0 (79.2–218.0)	117.3 (56.0–195.0)	***0.007***
Creatinine (mg/dl) median (range)	1.18 (0.76–1.48)	1.045 (0.78–1.60)	0.45
Hematocrit median (range)	40.9 (30.10–47.30)	40.75 (30.0–51.3)	0.98
Fibrinogen (mg/dl) median (range)	384.0 (240–557.0)	359.0 (213.0–650.0)	0.67
Leukocytes (mln/ml) median (range)	8.45 (6.55–11.60)	8.0 (5.1–13.0)	0.18
Lymphocytes (%) median (range)	22.8 (12.2–41.1)	24.95 (9.7–45.3)	0.82
Monocytes (%) median (range)	8.7 (5.4–14.2)	7.7 (4.5–14.8)	0.07
Neutrophils (%) median (range)	63.10 (46.3–81.6)	64.20 (44.50–82.30)	0.52

Data are presented as frequencies or median (minimum–maximum). Statistical significance for binary variables was assessed using generalized linear models, while metric values were analyzed using linear mixed regression models.
